# Built to last: Theta and delta changes in resting‐state EEG activity after regulating emotions

**DOI:** 10.1002/brb3.2597

**Published:** 2022-05-13

**Authors:** Gaia Lapomarda, Stefania Valer, Remo Job, Alessandro Grecucci

**Affiliations:** ^1^ Department of Psychology and Cognitive Sciences University of Trento, Trento, Italy; ^2^ Department of Psychology Science Division New York University of Abu Dhabi Abu Dhabi UAE

**Keywords:** delta frequency, distancing, emotion regulation, resting‐state EEG, theta frequency

## Abstract

**Background:**

: Over the past years, electroencephalography (EEG) studies focused on task‐related activity to characterize cortical responses associated with emotion regulation (ER), without exploring the possibility that regulating emotions can leave a trace in the brain by affecting its oscillatory activity. Demonstrating whether the effect of regulation alters the brain activity after the session and whether this reflects an increased cognitive regulatory ability has great relevance.

**Methods:**

: To address this issue, 5 min of electrical brain activity at rest were recorded before and after (1) one session in which participants perceived and regulated (through distancing) their emotions (regulation session, ReS), and (2) another session in which they only perceived emotions (attend session, AtS). One hundred and sixty visual stimuli were presented, and subjective ratings of valence and arousal of stimuli were recorded.

**Results:**

: Behavioral results showed the efficacy of the regulation strategy in modulating both arousal and valence. A cluster‐based permutation test on EEG data at rest revealed a significant increase in theta and delta activity after the ReS compared to the AtS, suggesting that regulating emotions can alter brain activity after the session.

**Conclusions:**

: These results allowed us to outline a comprehensive view of the neurophysiological mechanisms associated with ER, as well as some possible implications in psychotherapy.

## Graphical abstract

Graphical Abstract:

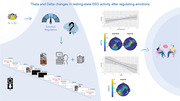

## 1 INTRODUCTION

The study of the neural mechanisms of emotion regulation (ER) is one of the cornerstones of contemporary affective neuroscience research. Emotions are powerful determinants of our daily life since they influence our thoughts, perceptions, and behaviors (Giorgetta et al., 2012; Grecucci et al., 2011; 2020). Thus, the ability to regulate emotions is essential to adapt to an ever‐changing environment (Ochsner & Gross, 2005). Its relevance stems also from clinical observations that reveal the association between difficulties in regulating emotions and several psychopathologies (Beauchiane and Zisner, 2017; Dadomo et al., 2016; 2018; De Panfilis et al., 2019; Frederickson et al., 2018; Grecucci et al., 2017; 2020; Kring and Sloan, 2009). For all these reasons, ER strategies have been recently incorporated into many psychological treatments (Grecucci et al., 2017; 2020; Messina et al., 2020).

Several studies aimed to identify the neural structures involved in ER using functional and structural magnetic resonance imaging (fMRI) (Buhle et al., 2014; Grecucci et al., 2013a, 2013b; Kohn et al., 2014; Ochsner & Gross, 2005; Pappaianni et al., 2019). Until recently, the temporal dynamics associated with the regulation of emotional stimuli were less addressed, although the field of affective neuroscience would benefit from a better temporal decomposition of the psychophysiological mechanisms involved in ER. Recent evidence, obtained in exploring the electrocortical activity by capitalizing on electroencephalography (EEG), reported reduced late positive potential (LPP) when regulating emotional stimuli was performed by applying the strategies of distraction (Paul et al., 2013), suppression (Moser et al., 2006; Gan et al., 2015), and reappraisal (Foti and Hajcak, 2008; Gan et al., 2015). LPP is a slow and long‐lasting positive deflection with a centroposterior distribution, which is larger for emotional than for neutral stimuli (Schupp et al., 2000). Qi and colleagues (2017) extended the above results to the use of detached reappraisal or distancing. More recently, also Grecucci and collaborators (2019) demonstrated a similar LPP modulation when participants used the strategy of distancing from unpleasant stimuli. All this evidence points toward the direction of a modulation of the LPP as a physiological signature of the effect of ER. From a psychological point of view, LPP seems to reflect increased directed attention and augmented working memory load, for better processing of salient stimuli (Schupp et al., 2000). Its modulation may indicate a decreased attentional and mnemonic effort when regulating emotions.

So far, we focused on the neurophysiological mechanisms associated with the effect of ER (the time window in which the emotional response elicited by the stimulus is regulated, Grecucci et al., 2019; Sulpizio et al., 2020). Few studies tried to investigate the neural mechanisms involved in the effect of applying the strategy to regulate emotions (ES, the time window when participants select/apply the strategy). In two fMRI studies, Grecucci and collaborators (2013a, 2013b) demonstrated a dissociation between the brain mechanisms subserving the ES, and those related to the ER. Specifically, strong activation of the dorsolateral prefrontal cortex (DLPFC) was associated with ES, whereas reduced activation of the insula, among other structures, was associated with ER. Moving to EEG studies, a recent experiment (Grecucci et al., 2019) reported an increase in negativity component over frontocentral electrodes for the ES, starting approximately 2.5 s after strategy presentation and lasting until the target appeared. This negativity was interpreted as a stimulus‐preceding negativity (SPN). SPN has been interpreted as reflecting increased attentional orienting to, and anticipation of, incoming stimuli to facilitate their elaboration or the execution of a task (Brunia, 2003; Brunia et al., 2012; Brunia & Van Boxtel, 2004).

In addition to the localizationist efforts offered by fMRI studies, and the temporal dynamics unfolding provided by event‐related potentials (ERPs) studies, another precious source of information for a better understanding of the mechanisms involved in ER is brain oscillatory activity (for a review see Knyazev, 2007). Brain oscillations can be highly informative on the brain's physiological and cognitive functioning subserving the processes under investigation (Sulpizio et al., 2020; Knyazev, 2007). Nonphase locked oscillatory responses provide insights on the functional network dynamics involved in cognitive processes (Varela et al., 2001; Knyazev, 2007). Thus, the processing units of interest can be better understood by looking at frequency changes rather than at localization (fMRI), or temporal unfolding (ERP). However, only a few studies focused on frequency characterization of ER. Ertl and colleagues (2013) found increased theta oscillations at frontal electrodes when participants reappraised emotional pictures. Uusberg and collaborators (2014) also showed increased theta power when distracted from emotional stimuli as compared with the neutral ones. However, these studies did not clearly separate the ES from the ER. A recent study by Sulpizio et al. (2020) addressed this issue more directly. When participants were asked to regulate the emotional reactions elicited by stimuli, a decrease in the theta and beta bands in posterior regions emerged (ER). Interestingly, when implementing the strategy (distancing, ES), an early increase in the theta band in posterior regions was observed (Sulpizio et al., 2020). This evidence supports the involvement of changes in theta oscillations in both these processes. Evidence has been reported for the involvement of theta frequency in a variety of behavioral and emotional variables (Knyazev, 2007), as well as in high‐level processes including cognitive control, inhibition, and sustained attention, known to be involved in voluntary top–down regulation strategies (Anderson et al., 2010; Sauseng et al., 2007). Notably, increased theta activity has also been documented when separate brain regions interact (Sauseng et al., 2007). For example, animal evidence showed coupling activity in the theta range between prefrontal control areas and subcortical emotion‐related regions (such as the amygdala) in the context of fear conditioning and extinction (Lestinget et al., 2011). This is especially relevant when considering that, in a typical ER task, there are regions implementing the strategy (ES) mainly lateral and mid frontal regions, and regions modulated after regulating (ER) as the amygdala and insula. According to this view, increased theta activity may reflect the combined work of modulating and modulated regions to successfully regulate emotional reactions.

Theta activity has been documented in expert meditators compared with nonmeditators (see Cahn et al., 2012 for a review). Indeed, after the regulation session (ReS), participants can experience a sense of increased wellness and relaxation from having their emotions regulated. Such feeling may be reflected in an increase in both theta and delta waves. Although there is no evidence of such effect during explicit ER tasks, increased delta waves have been found in the meditation literature (Harmony, 2013; Faber et al., 2008). This increased activity may correspond to the inhibitory activity of the medial prefrontal cortex that would lead to a reduction of emotional and cognitive engagement—described by the meditators as a state of distancing and detachment. A study by Tei and colleagues (2009) showed a significant increase in delta EEG frequency band (1.5−3.5 Hz) in a group of Qigong meditators compared with controls. This result may indicate the inhibition exerted by the prefrontal cortex and anterior cingulate cortex over emotionally related areas, as confirmed in another fMRI study on meditators (Hölzel et al., 2007).

An intriguing but yet not explored question is whether such frequency modulations are limited to the moment of the regulation (during the task), or whether they persist after the effort of regulation. If the effect lasts only a few seconds during the execution of the regulation process (as previously shown by ERP experiments), we may think that ER strategies are just effective when we use them, but do not hold the power to change our brain activity more permanently. On the contrary, if we demonstrate their long‐lasting effect, this may imply that the process of regulating emotions can leave a trace in the brain by changing its dynamics. This is of particular interest considering psychotherapy practice. During a therapy session, the therapist elicits problematic emotions related to the patients’ symptoms and helps to regulate them (Dadomo et al., 2016, 2018; Frederickson et al., 2018; Grecucci et al., 2020). One implicit assumption in psychotherapy is the potential extension and generalization to the daily life of the improvements conquered by the patients during the therapy sessions. One recent experiment showed that the effect of ER training is to reduce over time self‐reported negative affect after the training itself (Denny and Ochsner, 2014). We also know that therapies that incorporate cognitive regulation strategies can improve regulatory abilities and reduce anxiety and depression in daily life (Berking et al., 2008; 2013; Butler et al., 2006; Dobson, 2010). Demonstrating that, in a simplified but more controlled experimental setting, the application of regulation strategies can alter brain activity not only at the moment of the regulation (Sulpizio et al., 2020) but also later on, may represent the first evidence of the important effect the psychotherapy can have on the patients’ daily life. Discovering whether ER strategies change brain activity after the regulation task is not only helpful for clinical application but is of great interest for affective neuroscience as it may shed light on how affective processes affect brain functioning. Given that each frequency is associated with specific neurophysiologic mechanisms and has a psychological counterpart, investigating brain oscillations after regulating emotions can allow us to detect what happens in the brain after regulating emotions in terms of neurophysiologic mechanisms. One interesting question is whether the brain activity after ER can be ascribed to a decreased emotional activity (a better regulated emotional state, similar to an effect of regulation (EoR, Grecucci et al., 2013a, 2013b; 2017; 2019; Sulpizio et al., 2020), or an increased cognitive ability to top–down regulate future emotions (similar to an effect of strategy, ES). In a recent experiment (Sulpizio et al., 2020), it was found that during the implementation of the strategy, an early *increase in theta* band in posterior regions was observed (ES), whereas *a decrease in theta and beta* bands was observed when regulating the emotions elicited by visual stimuli (EoR). Thus, an intriguing question is whether the trace left by the ReS can be attributed to the maintenance of the ES or of the EoR.

The present study aims to test, for the first time, post‐ER effects on brain oscillatory activity. To this aim, we recorded 5 min of electrical brain activity at rest before and after two different sessions in which participants attended to (attend session, AtS) and regulated (ReS) emotions elicited by affective pictures, rating them in terms of arousal and valence. Specifically, we focused on a particular ER strategy, named distancing (Grecucci et al., 2013c, 2015a; Kross & Ayduk, 2017). This strategy consists in assuming a detached and nonjudgmental perspective as if the incoming stimulus has nothing or little to do with oneself. According to the literature, and based on the above considerations, we expect to observe a modulation of theta frequencies in the post‐ReS, as a reflection of ongoing regulatory processes that extend over the session. One hypothesis is that theta may be increased in the post‐session. This being the case, the results may be interpreted as an extension of the effect of strategy observed during the task execution (Grecucci et al., 2013a, 2013b; Grecucci et al., 2019; Sulpizio et al., 2020), as if the brain continues to train itself to improve regulatory abilities to better face future emotional stimuli. Such increased top–down control may also be reflected in an increase in delta activity. In particular, the strategy of distancing may increase delta activity at rest, similarly to what has been observed during and after meditative states (Harmony, 2013; Faber et al., 2008). Alternatively, we may find a decrease in theta frequencies similarly to the effect of regulation previously found by Sulpizio et al. (2020), which can be interpreted as a protracted regulated emotional state.

## 2 METHOD

### 2.1 Participants

Thirty‐five adults participated in the experiment. The data of two participants were discarded because of the high number of artifacts in the EEG data. The final sample was of 33 participants (16 female, mean age: 25.24, SD: 3.04). All participants were right‐handed, Italian native speakers, with normal or corrected‐to‐normal vision, with no history of psychiatric or neurologic problems. Participants were first informed about the nature of the study, and then they gave written informed consent to their participation. The Ethical Committee of the University of Trento approved the study.

### 2.2 Materials

The experiment comprised visual stimuli that participants were trained to attend to or to apply an ER strategy upon (distancing). Before starting the experimental procedure, an instance of a negative picture was presented, accompanied by an explanation of how to apply distancing.

#### 2.2.1 Stimuli

Picture stimuli were taken from the International Affective Picture System (Lang et al., 2008). Stimuli could be either neutral or negative according to their valence. Eighty stimuli per each category were selected, for a total of 160 pictures. The negative pictures had low valence (1.51–3.90, *M* = 2.59, SD = 0.64) and medium‐high arousal (5.21–7.07, *M* = 6.09, SD = 0.51). The neutral pictures had medium valence (4.14–6.65, *M* = 5.29, SD = 0.57) and low arousal (1.72–4.89, *M* = 3.09, SD = 0.68). Both neutral and negative pictures were divided into two subsamples and associated with the two experimental conditions (AtS vs. ReS). The two subsamples did not differ from each other, in either valence (*t* = 0.37, *p* = .90) or arousal (*t* = 0.52, *p* = .64).

### 2.3 Procedure

When the participants came to the laboratory, they were first asked to complete two questionnaires, ERQ and DERS. Then, 5 min resting‐state EEG (RS‐EEG) was recorded, to provide a baseline measure of brain activity before assessing any effect of regulating emotions. After that, participants completed the ER task (described below) consisting of two randomized sessions, each one followed by 5 min of RS‐EEG.

Each session of the task (AtS, ReS) lasted ∼9 min, with small fluctuations depending on the individual time required to rate the valence and arousal dimensions through the self‐assessment manikin (SAM). So, the whole experiment lasted ∼35 min (∼20 min for the ER task, 5 min RS‐EEG before starting the experiment, 5 min RS‐EEG after the first session, and 5 min RS‐EEG after the second session).

Finally, a debriefing was provided to explain the whole procedure and the aim of the study.

#### 2.3.1 Questionnaires

Before the experiment, participants were asked to fill out two questionnaires: the emotion regulation questionnaire (ERQ, Gross & John, 2003) and the difficulties in emotion regulation scale (DERS, Gratz & Roemer, 2004).

*Emotion regulation questionnaire*. The ERQ (Gross and John, 2003; Balzarotti et al., 2010) assesses individual differences in the use of reappraisal and expressive suppression in regulating emotions in everyday life. It includes a subscale for evaluating reappraisal (six items) and a subscale for suppression (four items). Higher scores reflect the more frequent use of a particular strategy.

*Difficulties in emotion regulation scale*. The DERS (Gratz & Roemer, 2004; Italian version of DERS, Sighinolfi, et al., 2010) measures clinically relevant difficulties in ER based on an extensive conceptualization of ER. It includes six subscales (emotion acceptance, goal‐directed behavior, impulse control, emotional awareness, flexibility in strategies, and emotional clarity), to control for dispositional attitudes that may help or hinder individuals from regulating their emotions. The higher the scores, the higher the levels of emotional dysregulation.

#### 2.3.2 ER task

The experiment consisted of two sessions, whose order was randomized across participants. In one session, participants were instructed to simply attend to the stimuli and experience the emotions elicited (AtS). In the other one, they actively regulated the elicited emotions by applying the regulation strategy, that is *distancing* (ReS). Before the experimental session, participants were provided with a written protocol describing how to apply the *distancing* strategy. In this protocol, participants were told to put themselves into a detached perspective, as if that event was far from their lives, and not connected with them. In a training, a negative picture was presented accompanied by an explanation of how to apply the strategy (Grecucci et al., 2019; Sulpizio et al., 2020). For the experimental session, the trial sequence was the following: after the first RS‐EEG recording (5 min), and before the first experimental session, a practice session composed of four trials—two per condition (AtS vs. ReS)—was presented. Then, instructions on the following session were given (AtS or ReS). A black screen with a fixation cross appeared for 2250 ms, and after that, a picture (stimulus) was projected for 3000 ms. To remind the participant of the current session, each stimulus was preceded by a shape: a circle to indicate the AtS, and a downward arrow to indicate the ReS (distancing). In each session, 80 stimuli were shown (half negative, half neutral) in random order, for a total of 160 stimuli in the two sessions. Specifically, always the same 40 negative and 40 neutral pictures were assigned to the AtS, and other different 40 negative and 40 neutral pictures were assigned to the ReS. Thus, specific neutral and negative stimuli were associated with the two experimental conditions (AtS vs. ReS), and they were balanced in terms of arousal (NEU: *p* = .18, NEG: *p* = .80) and valence (NEU: *p* = .73, NEG: *p* = .94).

**FIGURE 1:**
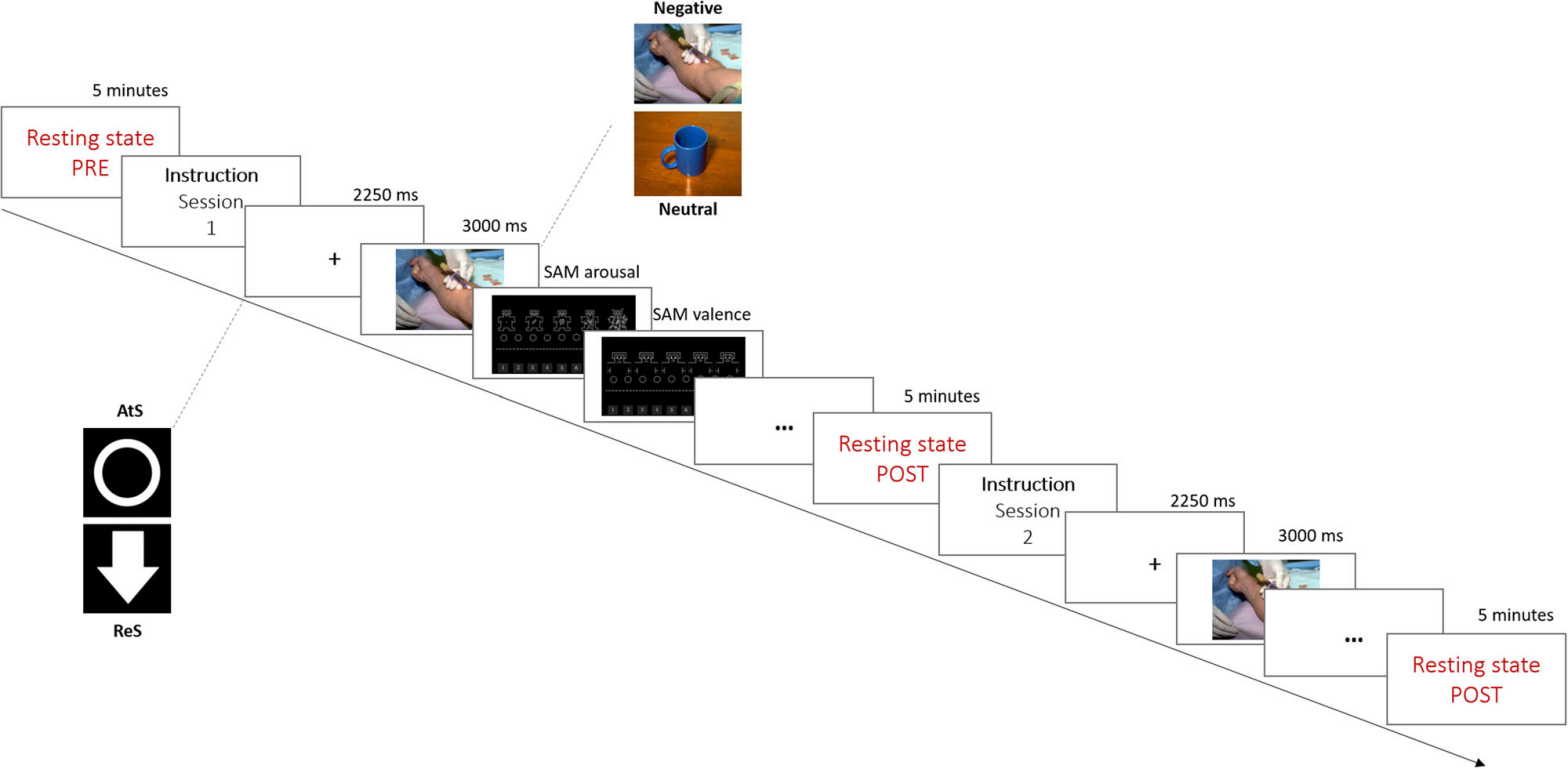
Timeline of events during the emotion regulation task

After each stimulus, participants rated their emotions on both the valence and arousal dimensions using the SAM procedure on a 9‐points Likert scale (Bradley & Lang, 1994; Bradley et al., 2007) (Figure 1). At the end of each session, 5 min of RS‐EEG were recorded.

#### 2.3.3 EEG recording

We recorded 5 min of electrical brain activity at rest (RS‐EEG) with eyes open (Barry, et al., 2007) at the beginning of the experiment to provide a baseline measure of brain activity, and after each of the two sessions, for a total of three recordings. The participants were comfortably seated in a quiet, mildly lit laboratory room. They were asked to minimize all their movements during the whole time of the recording.

EEG was recorded from 64 scalp electrodes (Fp1, Fpz, Fp2, AF7, AF3 AF4, AF8, F9, F7, F5, F3, F1, Fz, F2, F4, F6, F8, F10, FT7, FC5, FC3, FC1, FCz, FC2, FC4, FC6, FT8, T7, C5, C3, C1, Cz, C2, C4, C6, T8, TP7, CP5, CP3, CP1, CPz, CP2, CP4, CP6, TP8, TP10, P7, P5, P3, P1, Pz, P2, P4, P6, P8, PO7, PO3, POz, PO4, PO4, PO8, O1, Oz, O2) mounted on an elastic cap, positioned according to the international standard position (10–20 system) (Jasper, 1958). An additional external electrode was placed below the left eye (Ve1). All electrodes were referenced to the left mastoid (M1), and the ground was placed anteriorly to AFz. Impedance was kept below 10 kΩ. Data were acquired at the sampling rate of 1000 Hz and were filtered with a high‐pass filter (0.2 Hz cut‐off) and a low‐pass filter (100 Hz cut‐off).

### 2.4 Data reduction and statistical analyses

#### 2.4.1 EEG data

Offline preprocessing was performed using the FieldTrip toolbox for EEG/MEG analysis (Oostenveld et al., 2011; http://fieldtriptoolbox.org). After a careful visual inspection, we removed noisy channels from the data (de Cheveigne & Arzounian, 2018), and interpolated bad electrodes containing no useful brain signals (as suggested by Cohen, 2014). The signal was then corrected for eye blinks and ocular movements by inspecting independent component analysis components and manually removing artifactual data. On average, 20% of the epochs (out of 60 epochs per session) were rejected for each participant. Also, we interpolated no more than one electrode per participant. Data were re‐referenced to the common average. High‐pass and low‐pass filters were set at 0.2 and 100 Hz, respectively. Recordings were subsequently segmented into 60 segments of 5 s each. A fast Fourier transform (hamming window length 10%) was used to estimate spectral power density (μV2 /Hz) in the frequency bands (Putman, 2011).

#### 2.4.2 Statistical analyses

*Cluster‐based permutation analysis*. A nonparametric cluster‐based permutation approach was used (Maris & Oostenveld, 2007). This is a data‐driven comparison that considers all channels and all time points, providing appropriate control for multiple comparisons. In this method, comparisons between experimental conditions are performed for each sample using *t*‐tests. Values of *t* that exceed a predetermined threshold (*p* < .05) are clustered based on temporal and spatial adjacency, and cluster‐level statistics are calculated by summation of each cluster's *t*‐values. Then, a null distribution of the test statistics assuming no difference between conditions was approximated by generating 1000 random permutations of the observed data and computing the cluster‐level summed *t*‐values for each randomization (Groppe et al., 2011; Maris & Oostenveld, 2007).

*Correlation analyses*. To assess whether the individual tendency to regulate emotions in daily life and possible emotional difficulties were associated with regulation success, we conducted Spearman correlation analyses between ERQ and DERS subscales scores, and the regulation index (i.e., the differential rating scores of valence and arousal) given by the SAM procedure during the ER task. ERQ and DERS, as well as ratings of arousal and valence scores from SAM, were also correlated with neurophysiological results (i.e., the signal in different frequency bands after the cluster‐based permutation analysis).

## 3 RESULTS

### 3.1 ER task

**FIGURE 2:**
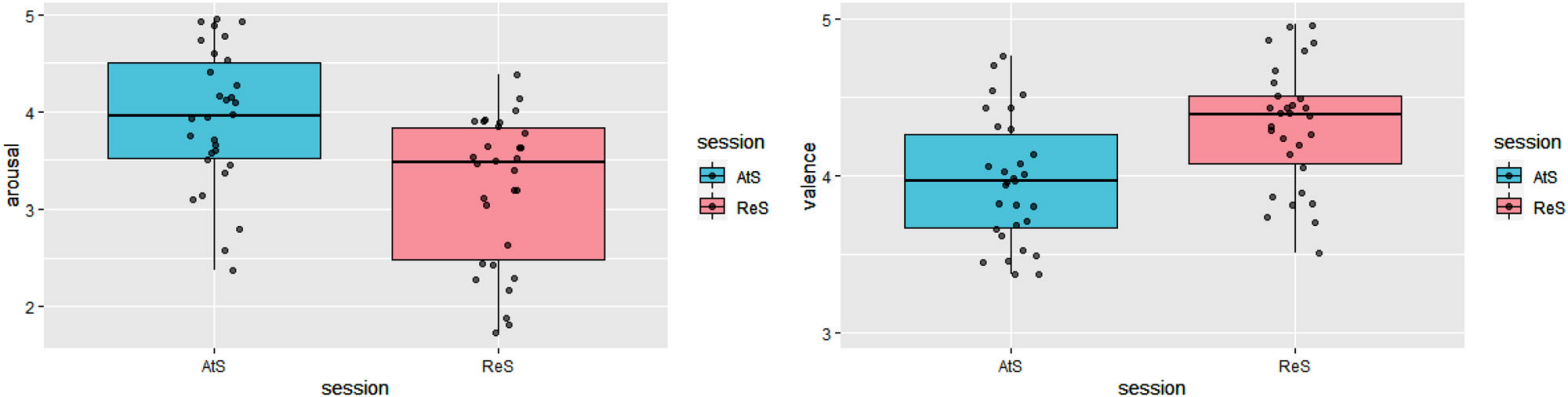
Behavioral results. Participants showed an effect of the regulation when applying the regulation strategy. Distancing (ReS) reduced arousal (strength of perceived emotions), and increased valence (perceived as less negative) with respect to the control condition Attend to (AtS). Error bars indicate the standard error of the mean

We first computed a paired sample *t*‐test on SAM ratings with all factors: index (arousal vs. valence) and regulation (distancing vs. attend). Analysis returned a significant effect of regulation, with diminished arousal (*t*(32) = −4.99, *p* < .001, *d *= −0.86) and increased valence (*t*(32) = 6.44, *p* < .001, *d *= 1.12) after regulating emotions. See Figure 2.

### 3.2 EEG data

#### 3.2.1 Cluster‐based permutation results

**FIGURE 3:**
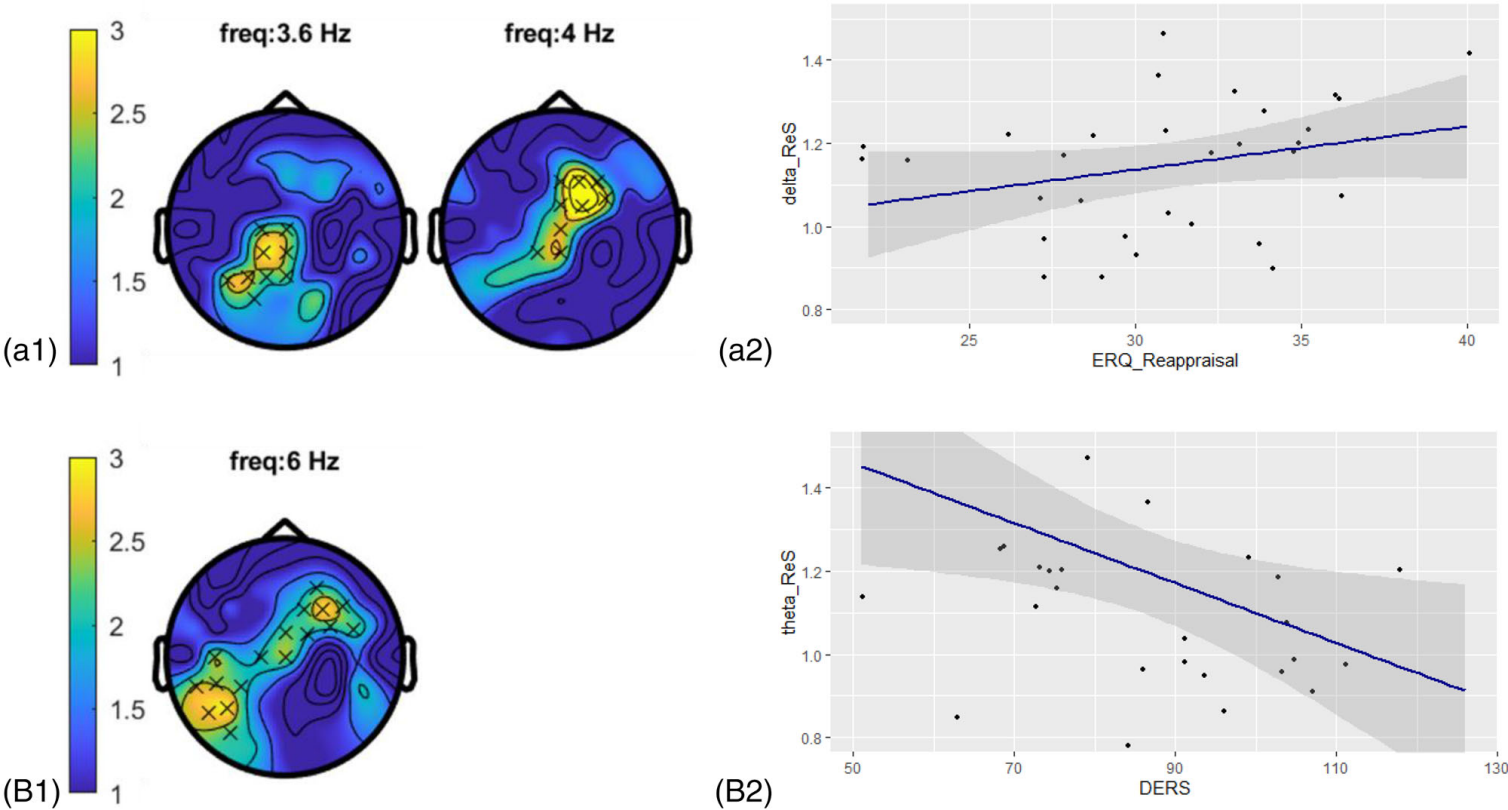
Topographic plots of cluster‐based permutation results and Correlation with ERQ and DERS scores. Topographic distributions of the positive clusters indicate an effect of condition corresponding to increased (A1) Delta and (B1) Theta frequencies in the post‐ReS compared with the post‐AtS. (A2) ERQ‐Reappraisal score positively correlated with *delta* activity after the regulation session (*r* = .43, *p* = .01), that is the better the tendency to regulate emotions, the higher the delta activity. (B2) DERS score negatively correlated with *theta* activity after regulation (*r* = −.41, *p* = .02), that is the stronger the difficulties in regulating emotions, the lower the theta activity

A nonparametric cluster‐based permutation analysis indicated an effect of condition (post‐ReS vs. post‐AtS), showing an increased RS‐EEG activity after regulating emotions. This corresponded to two positive clusters in the observed data (post‐ReS–post‐AtS), at the level of delta (3.6–4 Hz, *p* = .033) and theta (6 Hz, *p* = .040) frequencies. The maximum effect of 0.667 is observed on channel F2. See Figure 3.

#### 3.2.2 Correlation results

Neither ERQ nor DERS scores correlated with the ability to regulate emotions measured by arousal (ERQ‐reappraisal: *r* = −.14, *p* = .42, ERQ‐suppression: *r* = −.22, *p* = .21, DERS: *r* = −.26, *p* = .13) and valence (ERQ‐reappraisal: *r* = −.02, *p* = .89, ERQ‐suppression: *r* = .16, *p* = .35, DERS: *r* = 0.18, *p* = .31). The ERQ‐reappraisal score positively correlated with *delta* activity after both the ReS and the AtS (post‐ReS: *r* = .435, *p* = .011, post‐AtS: *r* = .402, *p* = .020, respectively; see Figure 3). Conversely, the ERQ‐suppression score did not show significant correlations with *delta* activity after any of the conditions (post‐ReS: *r* = .020, *p* = .912, post‐AtS: *r* = .124, *p* = .492). The DERS score negatively correlated with *theta* activity after regulation (*r* = −.401, *p* = .021; See Figure 3), but not after the AtS (*r *= −.089*, p *= .621). See Supplementary Materials. No other comparisons showed significant correlations (*p* > .05).

## 4 DISCUSSION

In the present study, we explored whether regulating emotions could affect brain oscillatory activity at rest after the session. We presented empirical evidence of a modulation of theta and delta frequencies after having regulated emotions as compared to the post control session. To do this, we recorded electrical brain activity at rest before and after one session in which participants perceived and regulated their emotions (post‐ReS) and another session in which they only attended to the emotions (post‐AtS). Cluster‐based permutation analysis on EEG data at rest revealed a significant increase in theta and delta activity in the post‐ReS compared to the post‐AtS. To control for the effectiveness of ER during the session, we computed the behavioral effect of regulation and found that distancing modulated both arousal and valence in the expected direction. In the next paragraphs, we discuss these findings.

### 4.1 Oscillatory activity after regulating emotions

One of the main aims of the present study was to extend previous psychophysiological results on the effect of ER strategies to the post‐ReS. So far, few experiments have focused on brain‐oscillatory activity during the execution of the regulatory task. Among these, Ertl and collaborators (2013) reported a significant increase in theta oscillations when participants applied reappraisal to downregulate emotions, and Uusberg and collaborators (2014) reported that distracting from emotional stimuli was related to increased theta power. In a recent study, Sulpizio and colleagues (2020) found that taking a detached perspective (applying distancing) from emotional situations increased theta activity in posterior regions during the implementation of the strategy (effect of strategy), in the attempt to top–down regulate emotions.

Notably, in our study, we found a similar increase in theta in the post‐ReS, as reported during the implementation of the strategy (Sulpizio et al., 2020). This pattern invites the conclusion that after the ReS the brain is in a similar state as when performing the task by deploying a strategy.

The interpretation of this finding is not straightforward, as theta frequency has been associated with a variety of behavioral, cognitive, and emotional tasks (Knyazev, 2007). Nevertheless, increasing evidence shows that theta frequency may play a role in multiple aspects of ER (Sulpizio et al., 2020). In other words, theta may be associated with multiple dimensions of the regulation process, from strategy implementation to regulation of emotional stimuli. We now discuss some possible physiological and psychological explanations of these findings, keeping in mind that different processes may cause similar oscillations’ modulation in a frequency band (Kappenman & Luck, 2011).

One possible systemic interpretation is that increased theta activity may reflect large‐scale interaction between cortical and subcortical regions (Sauseng et al., 2007). According to contemporary models of cognitive ER, different regions interact to produce the regulation (Grecucci et al., 2020; Ochsner and Gross, 2005). Such models stress the role of the prefrontal cortex (lateral and ventral parts) in interacting with subcortical areas responsible for the generation of emotions (amygdala, insula, etc.). Animal evidence may support this view by showing coupling activity in theta frequency between prefrontal control areas and subcortical emotion‐related regions (such as the amygdala) in the context of fear conditioning and extinction (Lestinget et al., 2011), two processes that may be involved in emotion generation and regulation (Grecucci et al., 2020). According to this view, increased theta activity may reflect the combined work of a large network of regions to successfully regulate emotions. In a similar vein, another line of evidence posits that theta changes are associated with cognitive control and inhibition (Anderson et al., 2010; Sauseng et al., 2007). This possibility extends the previous one by underlining the direction of the interaction between cortical and subcortical regions during the regulation process. Cognitive models of regulation assume that prefrontal areas exert a top–down modulation (and thus inhibit or decrease activity) of subcortical regions responsible for the emotional response.

Another interpretation of theta waves modulation (not necessarily alternative to the systemic‐inhibition hypothesis outlined above) is the one that links theta activity to meditative states (see Cahn & Polich, 2006, for a review of theta frequency and meditative states). Increased theta activity has been associated with proficiency in meditative practice (Aftanas & Golocheikine, 2001). Indeed, long‐term meditators exhibit higher theta activity relative to nonmeditators controls (Aftanas & Golocheikine, 2005; Andresen, 2000), and Qigong meditative technique seems to increase frontal midline theta activity (Pan et al., 1994), as well as mindfulness meditation (Dunn et al., 1999). Notably, frontal theta activity has been attributed to the activity of the anterior cingulate cortex, the medial and DLPFC (Asada et al., 1999; Ishii et al., 1999), all regions included in current models of ER (Grecucci et al., 2020).

Other interpretations may exist, related to a possible fatigue effect (Bernardi et al., 2015; Nelson et al., 2021). According to Bernardi and colleagues (2015), increased theta waves during situations of intense cognitive fatigue such as wakefulness may explain behavioral impairments (e.g., reduced control). Also, persistent changes in the theta range seem to reflect a form of neural fatigue (Nelson et al., 2021; Avvenuti et al., 2021). ER requires a certain amount of cognitive effort, but there is evidence of differences between strategies (Richards & Gross, 1999; Scheffel et al., 2021). For example, based on the process model of ER (Gross, 1998, 2014), suppression is cognitively demanding and effortful, as it consists in controlling emotional responses constantly (Richards & Gross, 2000). On the contrary, distancing is considered a less effortful strategy (Denny & Ochsner, 2014), because it occurs at an early stage of the emotion generation process. Indeed, due to its versatility and ease of deployment, distancing is a promising tool for clinical applications in a variety of affective disorders (Powers & LaBar, 2019). However, a direct comparison of ER strategies would shed light on whether different strategies require different amounts of cognitive effort, discriminating between distinct effort measures (Steele, 2020). Furthermore, the influence of situational factors and individual differences on subjective and physiological cognitive effort during ER is still unclear (Scheffel et al., 2021).

In addition to theta, we found an increase in delta frequencies after the ReS. In a few experiments, delta has been associated with emotional processing. For example, Putman and colleagues (2012) found an increased delta‐beta power coupling during the emotional Stroop task. Of note, participants who exhibited less emotional interference during the task also showed a larger coupling. Interestingly, words eliciting danger and usefulness seem to modulate oscillations in delta, theta, and low‐alpha frequency bands (Kryuchkova et al, 2012). These studies suggest an involvement of delta frequencies in emotional processing and regulation. Another line of evidence underlines the role of delta modulation in meditative‐like states. Although there is no previous evidence of delta modulation during explicit ER tasks, increased delta waves have been found in the meditation literature (Harmony, 2013; Faber et al., 2008). Another study by Tei and colleagues (2009) showed a significant increase in delta frequency (1.5−3.5 Hz) in a group of Qigong meditators compared with nonmeditators. This result may mirror the inhibition exerted by the prefrontal cortex and anterior cingulate cortex over emotionally related areas, as confirmed in another fMRI study on meditators (Hölzel et al., 2007).

Notably, theta activity in the post‐ReS correlated negatively with DERS. In other words, the higher the theta activity, the greater the awareness, the clarity, the acceptance, the inhibitory control, and the flexibility in using strategies to regulate emotions (all constructs measured by DERS). Thus, it seems that the post‐regulation oscillatory activity may be related to a more efficient mindset able to regulate future unpleasant events. Last but not least, delta activity correlated with reappraisal frequency usage (as measured by the ERQ‐reappraisal subscale), in both the post‐ReS and the post‐AtS conditions (although more strongly so for post‐ReS). However, due to the high number of comparisons, these results should be viewed and interpreted with caution.

### 4.2 Converging multiple neurophysiological processes behind ER processes

Besides the localizationist approach offered by functional magnetic studies, EEG‐based studies can capture the temporal dimension of the processes involved during ER, and more importantly, may allow a better understanding of the neurophysiological mechanism behind it. Building on the last three decades of experiments on emotion and, more recently, on ER, we have come to know the main ERP and the main frequency bands associated with multiple aspects of regulating emotions. We now succinctly outline these findings. During the time window when participants are instructed to use a strategy and prepare themselves to regulate the emotions elicited by subsequent stimuli (effect of strategy, ES), a SPN emerges (Grecucci et al., 2019). SPN is a slow and long‐lasting component occurring in anticipation of a variety of cognitive, attentive, and emotional task‐relevant stimuli (Brunia, 2003; Brunia et al., 2012; Brunia & Van Boxtel, 2004). Studies that focused on the time window of the strategy (even though they were not typically designed to separate the effect of strategy from the effect of regulation) found an increased SPN for reappraisal (Moser et al., 2009; 2017; Thiruchselvam et al., 2011), and for distancing (Grecucci et al., 2019) in the preimplementation period. This has been interpreted as enhanced recruitment of attentional resources or as a mindset to efficiently regulate subsequent relevant stimuli. LORETA source reconstruction indicates that the possible neural generators of this effect are the lateral and medial frontal cortex, but also the occipital cortex (Grecucci et al., 2019). This is in line with previous magnetic resonance studies about neural substrates of ER (Ochsner and Gross, 2005). During the ES, increased theta frequencies when reappraising (Ertl et al., 2013), distracting (Uusberg et al., 2014), and when distancing from emotional stimuli (Sulpizio et al., 2020) have been found when compared to the control “attend” condition. A greater modulation of theta frequency can indicate several processes related to a better systemic interaction between cortical and subcortical regions, possibly in the direction of inhibition of subcortical regions (responsible for emotion generation). These processes may underlie increased abilities to regulate emotions as shown in meditators and participants undergoing ER training. The present study extends these observations to the postregulation activity at rest, providing evidence that similar processes may take place during online regulation and after regulation.

In the time window when participants regulate the emotions elicited by stimuli (effect of regulation, ER), ERP evidence shows a modulation of the P300, also named LPP in the affective science literature. LPP is a positive slow wave with a posterior distribution, larger for emotional than neutral stimuli (Schupp et al., 2000). As an effect of regulation, this component has been found to be reduced independently of the strategy used (Moser et al., 2006; Foti & Hajcak, 2008, Schonfelder et al., 2014; Paul et al., 2013; Gan et al., 2015; Qi et al., 2017; Grecucci et al., 2019). The LPP is interpreted as enhanced processing of, and enhanced attention to, emotional salient stimuli, and indeed, it correlates with arousal (Cuthbert et al., 2000). Putative neural substrates of the LPP are the extrastriate visual system and emotion‐related structures such as the amygdala (Sabatinelli et al., 2007), and it may also reflect increased functional connectivity between occipital and frontal areas (Moratti et al., 2011). When considering effects other than ERP effects, in the time window when the regulation takes place, theta and beta frequencies decrease (Sulpizio et al., 2020; Tortella‐Feliu et al., 2014). Thus, lower activity in such bands may represent diminished salience and reduced attentional demand for the incoming emotional stimuli (Balconi & Lucchiari, 2006) as an effect of the application of the strategy to the emotions elicited by such stimuli (Aftanas et al., 2001; Guntekin & Basar, 2007; Woodruff et al., 2011). Nevertheless, Tolegenova and colleagues (2014) reported no effect in the beta band for participants asked to regulate their emotions while seeing negative movies. Thus, more evidence is needed to support the role of beta modulation during regulation. See Table 1 for an overview of EEG evidence from previous studies.

**TABLE 1:** Overview of EEG results from previous studies about emotion regulation

		Effect of strategy	Effect of regulation
		Implementation of strategy, preparatory mindset, anticipation of relevant stimuli	Modulation of emotional response, modulation of attention, semantic processes, working memory
During session	ERP evidence	↑ SPN	↓ P300
	ER‐TF evidence	↑ Theta	↓ Theta and beta
After session	RS evidence	↑ Theta and beta	

ERP, event‐related potentials; ER‐TF, event‐related time frequency; RS, resting state.

### 4.3 Limitations

Several limitations should be considered when interpreting the findings of the present study. First, we focused only on distancing, without considering other strategies. So, we cannot extend the present results to the usage of other strategies (e.g., reinterpretation, distraction, suppression). In a similar vein, our results are limited to the domain of affective pictures, and we do not know whether similar results could have been found using other stimuli (see, for example, Grecucci et al., 2019, for ERPs differences between affective words and pictures). Furthermore, as already highlighted, our correlational results must be interpreted with caution, due to the high number of comparisons.

## 5 CONCLUSION

In this study, we investigated oscillatory brain responses at rest in two conditions: one after participants regulated their emotions via the strategy “distancing”, and another in which they attended to the stimulus but did not regulate their emotions. The novel findings we report are that in the post‐ReS ‐ but not in the post “attend to only” session ‐ an increase in theta and delta frequencies occurs. Such an increase has been reported in previous studies during the time window when individuals are instructed to use a strategy and prepare themselves to regulate subsequent stimuli. A similar effect during and after the ReS can be interpreted as if the brain keeps training itself to prepare for regulating future stimuli. In other words, after regulating emotions the brain remains in a state of enhanced preparation for facing future emotional stimuli. This may lead us to hypothesize a relevant role of any post‐therapy session. During the session, the therapist helps the patient suffering from emotion dysregulation to increase her/his abilities to regulate past and future unpleasant events; after the session, the brain state allows patients increased access to, or capacity to put in place of, actual or possible mental actions related to ERs.

## FUNDING

This research did not receive any specific grant from funding agencies in the public, commercial, or not‐for‐profit sectors.

## CONFLICT OF INTEREST

All authors declare no conflicts of interest.

### PEER REVIEW

The peer review history for this article is available at https://publons.com/publon/10.1002/brb3.2597

## Supporting information

Supplement Material

## Data Availability

The data that support the findings of this study are available from the corresponding author, GL, upon reasonable request.
