# Supplementary material for: Built to last: Theta and delta changes in resting‐state EEG activity after regulating emotions
Source: Brain Behav. 2022 May 13;12(6):e2597. doi: 10.1002/brb3.2597 (PMC9226824; doi:10.1002/brb3.2597)
Supplement: Supplementary file 1 — Supplement Material [file BRB3-12-e2597-s001.docx]

**Supplementary Materials**


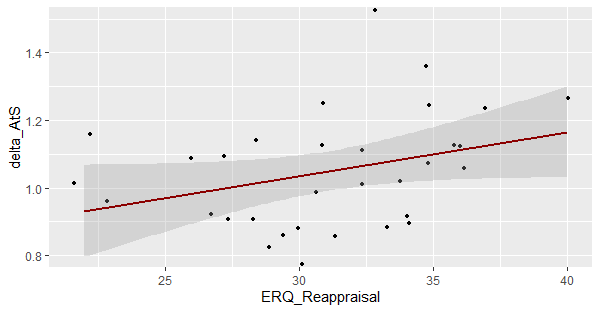


ERQ-Reappraisal score positively correlated with *delta* activity after the “attend to” session (*r = 0.402, p = .02*)


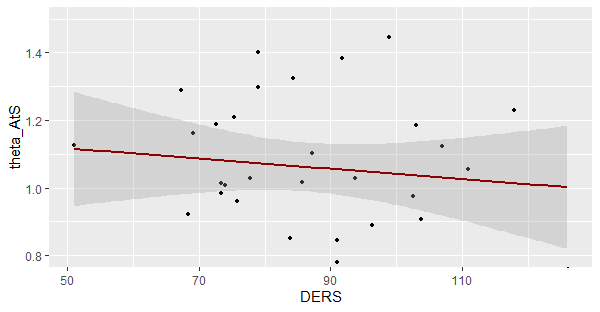


DERS score did not significantly correlate with *theta* activity after the “attend to” session (*r = -0.089, p = .62*)
